# An Unusual Case of Collagenous Gastritis: Incidental Finding in a Patient Presenting with Dysphagia

**DOI:** 10.1155/2019/5427085

**Published:** 2019-01-20

**Authors:** Chiu-Hsiang Liao, Maruf Saddik, Stephen Ip

**Affiliations:** ^1^Department of Pathology and Laboratory Medicine, University of Calgary, Calgary Lab Services, Canada; ^2^Department of Laboratory Medicine and Pathology, University of Alberta, Grey Nuns Hospital, Canada; ^3^Department of Gastroenterology, University of Alberta, Grey Nuns Hospital, Canada

## Abstract

Collagenous gastritis is a condition characterized by subepithelial deposition of collagen in the gastric mucosa. This condition is rare, with less than 100 cases reported in the literature. Patients with collagenous gastritis typically present with pain or diarrhea. Here, we present a case of a young woman with dysphagia who was found to have esophageal webs and an incidental finding of diffuse gastritis with a cobblestone appearance of the mucosa on endoscopy. Subsequent histology demonstrated features of collagenous gastritis, including mucosal inflammatory infiltrates and collagen deposition. This is one of the few case reports of incidental collagenous gastritis and highlights the importance of judicious use of biopsies.

## 1. Introduction

Collagenous gastritis (CG) is a condition characterized by the presence of mucosal inflammatory infiltrates and subepithelial deposition of collagen measuring greater than 10 micrometer. It was first described in 1989 by Colletti and Trainer in a 15-year-old patient who presented with recurrent abdominal pain and gastrointestinal bleeding [1]. Since then, less than 100 cases have been reported in the literature. There are two distinct clinical presentations of CG described by Lagorce-Pages and colleagues [2]. In the pediatric population, patients usually present with abdominal pain and anemia. In the adult population, patients present with chronic watery diarrhea with diffuse collagenous involvement of the gastrointestinal tract. Here, we present a rare case of CG presenting as an incidental finding on endoscopy for dysphagia.

## 2. Case Presentation

A 37-year-old female presented with a several year history of dysphagia to solid foods. She denied any symptoms of reflux or abdominal pain. She had been taking dexlansoprazole with no improvement in her symptoms. She also reported increased intake of nonsteroidal anti-inflammatory drug (NSAID) medications for a month prior to presentation. On physical examination, she appeared well. Her abdominal exam was unremarkable

**Figure 1:**
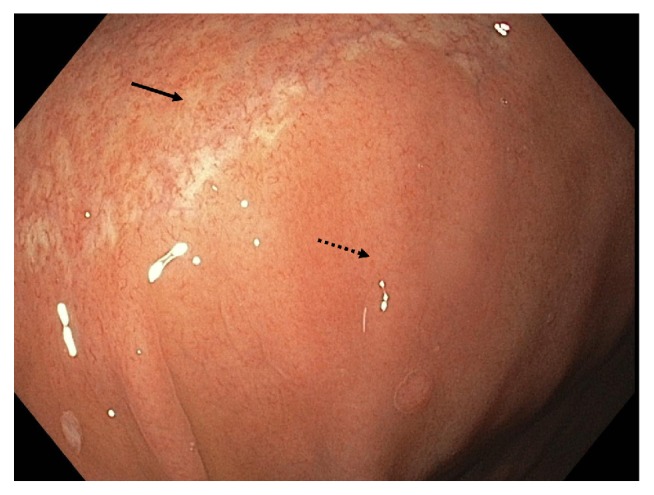
Endoscopic image of gastric mucosa showing an area of cobblestoning (solid arrow) and adjacent normal mucosa (dotted arrow).

**Figure 2:**
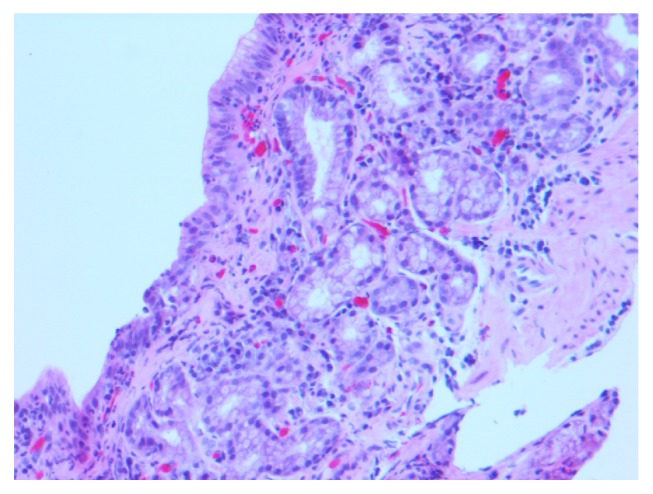
Histological section of gastric body showing focal mild atrophic change and area of subepithelial collagen deposit on hematoxylin and eosin stain. There is mild chronic gastritis. The surface epithelium is atrophic with intraepithelial neutrophils.

**Figure 3:**
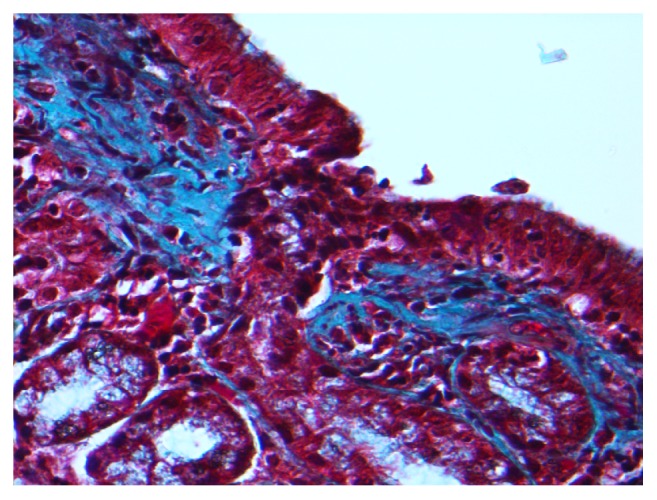
Histological section of gastric body showing area of subepithelial collagen deposition on trichrome stain.

Esophagogastroduodenoscopy (EGD) was performed and showed abnormal esophageal mucosa with two esophageal webs. There was also gastritis with vague nodularity in the gastric body (Figure 1). Esophageal biopsies showed no significant pathological abnormality. The stomach biopsies, taken from gastric body, showed mild chronic active gastritis with mild focal gastric atrophy and significant subepithelial collagen plate thickening. There were also entrapped inflammatory cells, red blood cells, and small capillaries compatible with CG (Figure 2). The thickened collagen plate was further highlighted by trichrome stain (Figure 3). The lamina propria was expanded, predominantly by plasma cells with admixed eosinophils and lymphocytes. The surface epithelium was atrophic with intraepithelial neutrophils. A Giemsa stain for* Helicobacter pylori* was negative and Congo red stain did not reveal any amyloid deposition. Serum protein electrophoresis did not show any evidence of a monoclonal protein and urine protein electrophoresis only showed minor albuminuria. Additional blood work, including celiac screen (anti-transglutaminase IgA: <1.0 U/ml, IgA: 2.36 g/L) and IgG4 titre (0.27 g/L), was all within normal limits. A complete blood count (CBC) performed one year prior to her presentation was normal (hemoglobin: 145 g/L, platelet: 216 × 10^9^/L, WBC: 6.4 × 10^9^/L). A diagnosis of CG was made. No new treatment was initiated and it was elected that she remains on the same dose of dexlansoprazole.

Subsequent upper and lower endoscopy were performed to rule out collagenous disease elsewhere. Colonoscopy was normal. EGD showed presence of friable tissue and multiple rings in the esophagus. There was mucosal cobblestoning and friable tissue in the gastric body. Biopsies of the duodenum, ileum, and colon were unremarkable. Biopsies of the gastric body showed minimal chronic inflammatory cell infiltrates and prominent mucosal lymphoid aggregates. Subsequent barium swallow demonstrated gastroesophageal junction outlet obstruction, for which the patient received esophageal dilation. Repeat EGD on follow-up 6 months later showed persistent esophageal webs and gastric mucosal cobblestoning with histological evidence of CG. In this instance, the gastric body mucosa had moderate chronic active gastritis with increased fibrosis beneath the surface epithelium and within the lamina propria, and the gastric antral mucosa showed a mild increase in fibrosis of the lamina propria.* H. pylori* was not detected in any of the biopsies taken.

## 3. Discussion

CG is a rare entity and the pathophysiology of this disease is still poorly understood. There are two classically described distinct clinical presentations of CG [2, 3]. Several atypical presentations of CG have also been reported, with gastric perforation being the most severe presentation reported thus far [4]. CG as an incidental finding in an asymptomatic patient is rare, first reported in a case series by Lagorce-Pages et al. [2] and subsequently reported in a later case series by Arnason and colleagues [3]. Our patient presented with dysphagia, but her symptoms could be explained by the presence of esophageal webs, likely related to NSAID use. She did not have any abdominal pain, dyspepsia, or other symptoms to suggest a primary gastric pathology. The histologic finding of CG likely does not explain her clinical presentation.

The first repeat EGD and gastric biopsies in our patient while receiving no new therapy showed no significant pathologic findings apart from minimal chronic inflammation despite endoscopic mucosal cobblestoning on endoscopy. While the endoscopic appearance of collagenous gastritis is classically described as mucosal erythema and cobblestoning, the sensitivity of endoscopic diagnosis of CG remains unknown. As previously described [2], collagen deposition in CG occurs preferentially in the depressed area of cobblestoning; therefore, the histopathological changes associated with CG may have not been adequately sampled by our biopsy site selection. The second repeat EGD performed in follow-up showed persistent gastric mucosal cobblestoning with biopsy-confirmed CG. This finding highlights the importance of proper sampling in making a histopathological diagnosis of CG, as it is unlikely for the CG to have healed and then recurred.

In our case of incidental CG, the patient had endoscopically abnormal-appearing mucosa with nodularity that warranted a biopsy to rule out a more sinister etiology. However, the pathological diagnosis of CG led to further investigations that did not ultimately change the medical management of this patient. In studies comparing the diagnostic yield between EGD performed with a clinically relevant indication to those without, it was shown that the diagnostic rate was significantly higher in patients with pertinent clinical indications for biopsy [5, 6]. Furthermore, there is a high discordance rate between endoscopic and pathological correlation with EGD. A study looking at correlation of endoscopic and pathologic findings showed that there is a discordance rate of 34% for patients with gastritis [7]; 41% of the discordant cases had normal endoscopy with abnormal pathology and 59% of the discordant cases had abnormal endoscopy with normal pathology [7]. Given the high discordance rate, the general recommendation is to not collect a biopsy sample of an area of suspected gastritis, in the absence of clinical symptoms [8].

In summary, we present a case of CG as an incidental finding in a patient with dysphagia. This is a rare entity that is most commonly symptomatic at presentation. To the best of our knowledge, this is the third reported case of incidental CG in an asymptomatic patient or patient presenting with unrelated symptoms. Our case report highlights the importance of considering the clinical indications when choosing whether to biopsy patients. In our case, obtaining gastric biopsies was necessary given the unusual cobblestone appearance on endoscopy. However, the patient's clinical management was unchanged after extensive investigations to rule out other causes of this histologic finding and to determine the extent of disease within the GI tract. Biopsy results need to be carefully interpreted in the context of a patient's clinical presentation.
